# Efficacy of Bioenergetic Health Index to Predict Delirium After Major Abdominal Surgery in Elderly Patients: A Protocol for a Prospective Observational Cohort Study

**DOI:** 10.3389/fmed.2022.809335

**Published:** 2022-04-25

**Authors:** Yi Zhao, Juan Liu, Mengchan Ou, Xuechao Hao

**Affiliations:** ^1^Department of Anesthesiology, West China Hospital, Sichuan University, Chengdu, China; ^2^The Research Units of West China (2018RU012), Chinese Academy of Medical Sciences, West China Hospital, Sichuan University, Chengdu, China

**Keywords:** postoperative delirium, elderly patients, BHI, mitochondrial function, major abdominal surgery

## Abstract

**Introduction:**

Postoperative delirium (POD) is a common disorder following surgery, which seriously threatens the quality of patients’ life, especially the older people. The multifactorial manner of this syndrome has made it hard to define an ideal method to predict individual risk. Mitochondria play a key role in the process of POD, which include inflammatory on the brain caused by surgeries and aging related neurodegeneration. As BHI (Bioenergetic Health Index) could be calculated in cells isolated from an individual’s blood to represent the patient’s composite mitochondrial statue, we hypotheses that HBI of monocytes isolated from individual’s peripheral blood can predict POD after major non-cardiac surgery in elderly patients.

**Methods and Analysis:**

This is a prospective, observational single-blinded study in a single center. 124 patients aged ≥ 65 years and scheduled for major abdominal surgery (>3 h) under general anesthesia will be enrolled. Preoperative and postoperative delirium will be assessed by trained members using Confusion Assessment Method (CAM). For patients unable to speak in the ICU after the surgery, Confusion Assessment Method for the Intensive Care Unit (CAM-ICU) will be used. All patients will undergo venous blood sampling twice to measure BHI (1–2 tubes, 5 ml/tube): before the surgery and 1 day after surgery in wards. After discharge, patients will be contacted by telephone 30 days after surgery to confirm the incidence of post-discharge complications. The severity of complications will be categorized as mild, moderate, severe or fatal using a modified Clavien-Dindo Classification (CDC) scheme.

**Ethics and Dissemination:**

The study has been approved by the Ethics Committee on Biomedical Research, West China Hospital of Sichuan University, Sichuan, China (Chairperson Prof Shaolin Deng, No. 2021-502). Study data will be disseminated in manuscripts submitted to peer-reviewed medical journals as well as in abstracts submitted to congresses.

**Clinical Trial Registration:**

[www.ClinicalTrials.gov], identifier [ChiCTR2100047554].

## Summary

1. To the best of our knowledge, no cohort study describing association between mitochondrial function and postoperative complications, and findings may promote clinical use of mitochondrial tests.
2. This is the first study to connect the perioperative mitochondrial function of monocytes isolated from an individual’s blood with POD.
3. This study was a single-center study and the sample size was relatively small.

## Introduction

### Background

Postoperative delirium (POD) is a cognitive disturbance characterized by acute and fluctuating impairment in attention and awareness. It commonly occurs between postoperative days 2–5. In the general surgical population, the incidence of postoperative delirium is reported to be 2.5–3% (1, 2). In patients aged more than 60–70 years, the incidence of postoperative delirium is considerably higher at 10–20% (3–5). Advancing age with decreased reserve and changes to the physical characteristics of the brain, including cerebral atrophy and changes in white matter, was associated with an increased risk of developing delirium (6–8). The severity of POD varies from individual to individual. Cognitive function changes may last for a few days, months or years, and there is a possibility of further deterioration (9). The occurrence of postoperative delirium lengthens hospital stay by 2–3 days and ICU stay by 2 days (10–12). Postoperative delirium is also associated with a 30-day mortality of 7–10%, compared with 1% in those without delirium (11–13).

Early identification of patients at high risk for delirium before surgery aids the clinician and allows for the development of strategies targeted at minimizing the occurrence of delirium. However, the multifactorial occurrence of POD makes it hard to foresee delirium even in fields with high incidence like cardiac surgery (14), and the tools for the prediction of delirium have shown to be insufficient (15). Potential blood markers can be divided into two categories: proinflammatory proteins and neuronal cell damage markers. Proinflammatory proteins are highly sensitive while with low specificity for the diagnosis or prediction of delirium, including IL-1, IL-6, C-reactive protein (CRP), interleukin, procalcitonin (PCT), and TNFa (16). Neuronal cell damage markers like protein S-100 and neuronal specific enolase (NSE) can only indicate an already ongoing detrimental process without any predictive ability. Beside blood markers, numerous risk prediction models are available for predicting delirium, including Katznelson, the original PRE-DELIRIC, and the international recalibrated PRE-DELIRIC model (13, 15, 17). But these models were formulated based on identified risk factors for patients in ICU (17). Validated prediction model for surgical patients is still lacking. All these limitations point to the need for better alternatives to assess preoperative risk of POD.

Understanding the potential role of mitochondria in POD pathophysiology is of importance due to the increase in our understanding of aging and inflammation. As cellular bioenergetics factories, the primary function of mitochondria is to generate ATP through electron transport chain and oxidative phosphorylation; however, this process is also accompanied by generation and release of reactive oxygen and nitrogen species (ROS, RNS) (18). Accumulation of ROS and oxidative damage is one of the cellular hallmarks of aging. As an extension of the free radical theory, the mitochondrial vicious cycle theory of aging emphasizes and refines the central role of mitochondria in the aging process (19). On the other hand, mounting evidence indicates that systemic inflammation on the brain caused by surgeries is the key process of the pathogenesis of POD (20). The impact of systemic inflammation on the brain can be profound. Neurons are highly vulnerable to the harmful effects of these reactive species due to its high metabolic rate, the predominance of fatty acids with a tendency to peroxidation, high intracellular concentrations of transition metals capable of catalyzing the formation of reactive oxygen species (ROS), and low levels of antioxidants (21). Systemic oxidative stress was associated with blood–brain barrier disruption and increased odds of developing postoperative delirium (22).

In central nervous system (CNS), mitochondrial dysfunction characterized by a progressive accumulation of mtDNA mutations, increased ROS production and impaired mitochondrial respiration, all of which have been shown to impair adult neurogenesis (23, 24) and result in neurodegenerative diseases (25, 26). Studies have proven that mitochondrial dysfunction is connected with neurodegenerative diseases, particularly in Parkinson’s disease (PD) (26), Alzheimer’s disease (AD) (25) and dementia. As an acute cognitive impairment, POD is closely related to these diseases in molecular pathways (27), which indicate us mitochondrial dysfunction also act as a key role in POD. And animal studies of elderly rats did prove mitochondrial dysfunction and oxidative stress contributes to the process of POD (28). In addition to be a main source of ROS, mitochondria are the prime targets of oxidative damage, which in turn reduces mitochondrial efficiency and leads to the generation of more ROS in a vicious self-destructive cycle (28, 29). Especially for elderly patients with chronic comorbidities, the pre-existing mitochondrial dysfunction make them more sensitive to perioperative changes (30). These findings suggest us mitochondria is the connection of inflammation and POD and the assessment of mitochondrial function may provide us with a clue to predict postoperative outcomes in aging patients.

Traditionally, the test of brain mitochondrial function needs fresh tissue samples, which limits its clinical use. However, the BHI (Bioenergetic Health Index), which proposed by Chacko et al., could be calculated in cells isolated from an individual’s blood to represent the patient’s composite mitochondrial profile (31). This advantage makes it possible for BHI developing to be a clinical test. Platelets, lymphocytes and monocytes are exposed to many soluble circulatory factors associated with metabolic stress and are, therefore, an ideal surrogate for determination of BHI in patients. Among which, monocytes are phagocytic cells which survey the body for sites of inflammation and play an essential role in the innate immune system (32–34). The clinical test carried out by Chacko et al. did prove that BHI was a sensitive measure of oxidative stress in human monocytes (35). Then, their following clinical study demonstrated that the BHI is significantly depressed in monocytes isolated from the post-operative pericardial fluid and blood of patients undergoing cardiac surgery (36). These findings suggest BHI is a functional biomarker of the impact of perioperative systemic oxidative stress. In brief, the BHI of monocytes in peripheral blood can reflect brain mitochondrial function and surgical systemic oxidative stress. Thus, we suppose the BHI can serve as a marker or predictor of POD. As there is no study to confirm the relationship between them, we conducted this study to test if HBI of monocytes isolated from individual’s peripheral blood can predict delirium after major non-cardiac surgery in elderly patients.

### Hypothesis and Objectives

We hypothesis that poor mitochondrial function with aging process makes brain vulnerable to systemic inflammation by surgeries. As BHI of monocytes in peripheral blood can reflect brain mitochondrial function and surgical systemic oxidative stress, we assume patients with low BHI have higher risk of POD.

The main objective of the current study was to find whether HBI of monocytes isolated from individual’s peripheral blood (preoperative or postoperative) can predict delirium after major non-cardiac surgery in elderly patients during hospitalization. The secondary objective was to determine whether BHI can predict moderate or severe complications within 30 days after surgery, because these events are also associated with perioperative inflammation/oxidative stress and poor organ function.

## Methods and Analysis

### Trial Design

**FIGURE 1:**
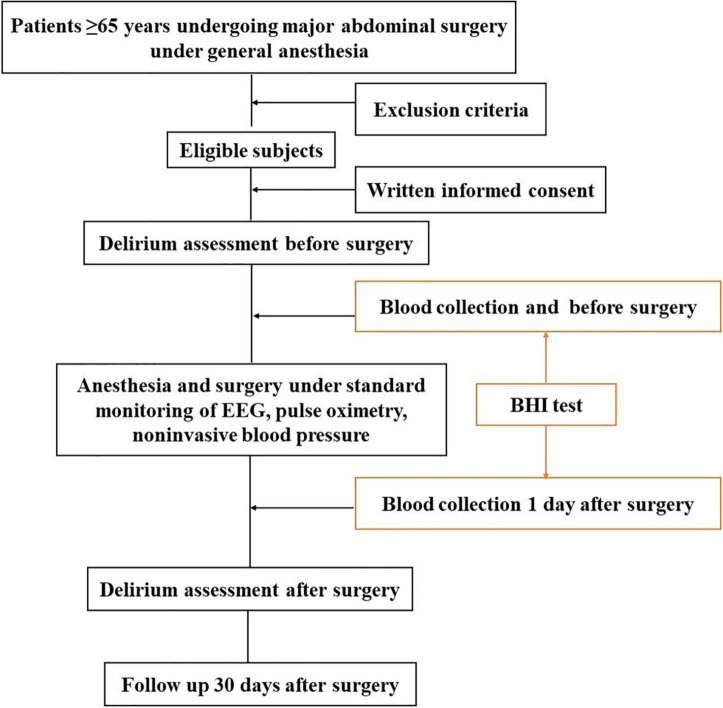
Flowchart of the study.

This is a prospective, observational single-blinded study being conducted in a single center. The flowchart of this trial is in Figure 1. The study will include patients scheduled for major abdominal surgery (> 3 h) under general anesthesia in the operating center of West China Hospital of Sichuan University, Chengdu, China from July 2021 to December 2021.

### Ethics Approvals and Registration

Ethic approval for this study has been obtained from the Ethics Committee on Biomedical Research, West China Hospital of Sichuan University, Sichuan, China (Chairperson Prof Shaolin Deng, No. 2021-502). The trial was registered at Chinese Clinical Trial Registry (ChiCTR2100047554). Participating sites obtained written informed consent by participating patients or their legal surrogates in accordance with local/national legislations. Fully data will be upload on the Clinical Trial Management Public Platform.

### Recruitment of Patients and Blinding

The patients will be recruited from the department of surgical ward in West China Hospital of Sichuan University. Patients undergoing major abdominal surgery under general anesthesia will be included in the study after obtaining informed consent. Before the surgery, investigators will identify suitable patients and approach them by telephone between 1 day and 1 week before surgery. Informed consents will be provided and interpreted by trained investigators, and they will be informed that their peripheral blood samples will be taken in this study. Once participants signed the consents, they will be enrolled and anonymized by an identification code. The subjects can voluntarily withdraw from the trial at any time. The BHI tests will be carried out by a skilled investigator. Preoperative delirium assessment and postoperative follow-up will be performed by trained members of the research team respectively, and they will be blinded to the outcomes of each other. Patients will be free to withdraw from the study at any time for any reason without prejudice to future care, and with no obligation to provide the reason for withdrawal.

***Inclusion criteria:*** (1) patients aged over 65 years, (2) no limitation of gender, (3) American Society of Anesthesiologists (ASA) physical status I–III, (4) mentally competent, (5) Informed and written consent.

***Exclusion criteria:*** (1) known hematological diseases, (2) long term use of sedative or psychotropic drugs, (3) unstable mental state, (4) history of allergy to anesthetics, (5) with learning difficulties, or mentally handicapped, (6) hemodynamic instability, (7) organ transplant surgery.

### The Bioenergetic Health Index Test

#### Blood Collection

All patients will undergo venous blood sampling twice to measure BHI (1–2 tubes, 5 ml/tube): before the surgery in the operating room and 1 day after surgery in wards. Blood was collected in vacutainers (BD Biosciences) containing 1.5 ml of ACD (acid citrate dextrose) solution (trisodium citrate, 22.0 g/l; citric acid, 8.0 g/l and dextrose 24.5 g/l). Blood samples need to be preserved temporarily at 4^°^C and send to the laboratory within 30 min.

#### Monocyte Isolation From Blood

Centrifuge the blood at 500 g for 10 min at room temperature. Then, the leucocyte-enriched layer on top of the RBC (red blood cell) pellet (buffy coat) will be diluted (1:4) with basal RPMI (Roswell Park Memorial Institute) media and applied to a Histopaque density gradient (specific gravity = 1.077/1.119, at room temperature, Sigma Chemical Co.) and centrifuged without application of the brake at 700 g for 30 min. then collect the peripheral blood mononuclear cells (PBMCs).

#### Bioenergetic Assessment of Monocytes

Determination of cellular bioenergetics will be performed after plating the cells on 8-well polystyrene plates designed for the extracellular flux (XF) analyzer (37). The number of PBMCs will be counted on Counstar automatic cell counter (IC 100, Shanghai Ruiyu Biotechnology Co.). Twenty microliter trypan blue will be added into the cell samples to get the number of living and dead cells, respectively. Researchers will resuspend purified monocytes and plate the diluted cells (300,000 living cells/well) in 200 μl on CellTak (BD Biosciences) coated assay plates and allow it to attach for 30 min at 37^°^C in a non-CO_2_ incubator. The cellular bioenergetics of the isolated cells will be determined using the XF analyzer (Seahorse Bioscience) in combination with the mitochondrial stress test (38). Real-time, non-invasive measurements of oxygen consumption rate (OCR) and extracellular acidification rate (ECAR) will be measured and correlated to mitochondrial function and glycolysis, respectively. The percent of non-mitochondrial and glycolytic monocyte OCR and ECAR will be assessed in the XF96 XF analyzer (Seahorse Bioscience) as a glucose stress test with sequential injections of glucose (5 mM), oligomycin (1.0 μg/ml) and 2-deoxyglucose (100 mM). Using the mitochondrial stress test protocol, inhibitors of the mitochondrial respiratory chain will be injected sequentially to assess the respiratory parameters: basal OCR, ATP-linked OCR, proton leak, maximal, and non-mitochondrial OCR. Reserve capacity will be calculated by the subtraction of basal OCR from maximal OCR. The optimum concentration of the inhibitors and activators to be used for the assessment of mitochondrial function were determined, as previously described (37, 39). The BHI was calculated using the following equation: (Reserve capacity × ATP-linked OCR)/(Proton leak × Non-mitochondrial OCR)^32^.

### Anesthesia and Surgical Procedure

All patients will undergo a standard preoperative assessment, including medical history, physical examination, laboratory blood tests, and a 12-lead ECG. If further investigations are necessary, they will be carried out at the discretion of the operating team. One day before surgery, responsible anesthetists will be asked to make a pre-operative subjective assessment of patients. Comorbidities are based on the medical history and self-reporting. Hypertension is considered to be present if the patient had previous medical documentation of hypertension and is currently taking antihypertensive medication. Coronary artery disease is defined as a history of angina; myocardial infarction; positive exercise, a nuclear or echocardiographic stress test; resting wall motion abnormalities on echocardiogram; coronary angiography with evidence of ≥ 50% vessel stenosis; or an electrocardiogram with pathologic Q-waves in 2 contiguous leads. A requirement for insulin or oral hypoglycemic therapy at the time of admission for surgery is considered to be diabetes mellitus. Arrhythmias are considered to be any type of previously diagnosed arrhythmias or abnormal ECG after admission. A patient will be considered a smoker, if they had a history of smoking within 1 year before surgery. Chronic obstructive pulmonary disease (COPD) was extracted from the patient’s primary care records and past medical history. Cerebrovascular disease was defined as a previous cerebrovascular accident or transient ischemic attack.

On the operation day, standard monitoring of electrocardiogram, pulse oximetry, non-invasive blood pressure and the bispectral index (BIS, Covidien LLC, MA, United States) will be performed throughout anesthesia to detect clinical vital signs. General anesthesia will be induced by intravenous propofol 1.5–2.5 mg/kg, and sufentanil 3 μg/kg, cisatracurium 0.2 mg/kg or vecuronium bromide 0.1 mg/kg subsequently administered. Anesthesia will be maintained with an intravenous remifentanil infusion and sevoflurane or desflurane inhalation or propofol TCI. After the surgery, patients will be transferred to the post-anesthesia care unit (PACU) or ICU.

### Outcome Measures

#### Primary Outcome

Trained members of the research team will assess patients for delirium use the Confusion Assessment Method (CAM) (40) or the Confusion Assessment Method for the Intensive Care Unit (CAM-ICU) (41) for patients who are unable to speak (e.g., still intubated) in the ICU. These methods (CAM and the CAM-ICU) are reliable and have been consistent with the Diagnostic and Statistical Manual of Mental Disorders, 4th edition diagnostic criteria for delirium (42). The severity of delirium will be assessed by the maximum daily score of the CAM-S, a severity scale for patients who screen positive for delirium based on the CAM.

Before the surgery, investigators will identify suitable patients and approach them by telephone between 1 day and 1 week before surgery. Those who consented will be performed delirium assessments 1 day before surgery. After the surgery, delirium assessments were done when patients could be aroused sufficiently (Richmond Agitation and Sedation Score –3 or higher) (43). Investigators will assess delirium twice per day from the first to the third postoperative day in the morning and afternoon. The new onset of delirium after the third postoperative day will be assumed to be unrelated to anesthetic or other intraoperative factors.

#### Secondary Outcome

Besides delirium assessments, other complications after surgery will also be recorded by trained members during hospitalization. After discharge, patients will be contacted by telephone 30 days after surgery to confirm the incidence of post-discharge complications. The severity of complications will be categorized as mild, moderate, severe or fatal using a modified Clavien-Dindo Classification (CDC) scheme.

### Data Collection

Data collected in the study are listed in Table 1. Electronic data will be stored and saved on a pass-word-protected computer. Hard (paper) copies of the consent form, questionnaire, and data sheets will be stored in a locked filing cabinet in the principal researcher’s computer. Downloaded from and members of the research team will be given access to the data. After the study, the data will be uploaded on the website of Chinese Clinical Trial Registry. As this is an observational study, data monitoring committee is not needed.

**TABLE 1:** Data collected in the study.

Type of data	Variables
Demographics variables	Age, Sex, Height, Weight, BMI
Comorbidities	ASA status, Motion equivalent, Comorbidities (Hypertension, Coronary artery diseases, Arrhythmias, Diabetes mellitus, Current or recent smoker, COPD, Cerebrovascular disease)
Procedure type	Hepatobiliary, Gastrointestinal, Pancreatic
POD assessment	CAM or CAM-ICU scores
BHI	Real-time, non-invasive measurements of OCR and ECAR
Postoperative complications	Events occurring until 30 days after hospital discharge

*COPD, Chronic obstructive pulmonary disease; ASA status, American Society of Anesthesiologists Physical Status; MET, Metabolic equivalent; CAM, Confusion Assessment Method; CAM-ICU, Confusion Assessment Method for the Intensive Care Unit; OCR, oxygen consumption rate; ECAR, extracellular acidification rate.*

### Sample Size and Statistical Analysis

The sample size calculation was based on comparing the area under the curve (AUC) of the receiver operating characteristic (ROC) curves using MedCalc (Version 19). Assuming an outcome event rate of 9–11% (based on our previous study), a moderately good AUC of 0.8 for the BHI, a sample size of 112 patients had 90% power to detect this clinically relevant difference in AUC values (2-sided alpha of 0.05). To account for 10% of patients who may have been lost to follow-up, we aimed to recruit a total of 124 patients to the study. When the study finished, the missing data will be excluded.

Statistical analysis was performed using SPSS Version 15 software (SPSS, Chicago, IL, US). All statistical tests were 2 sided and significance was assumed at *P* < 0.05. Comparison of interval data was performed using independent sample *t*-tests, Mann–Whitney or Kruskal–Wallis tests when appropriate. The ROC curve analysis was performed to identify the predictive values of the BHI for POD and postoperative complications. The performance of continuous variables was analyzed with ROC curves, with the area under the curve (AUC) being calculated. Cutoff values were evaluated based on the Youden index. Relative risk (RR) and 95% confidence intervals (CIs) were estimated using a chi-squared test.

### Adverse Events and Serious Adverse Events

This is a prospective observational clinical trial and the only intervention on patients is vein puncture for blood collection. All drugs administered during anesthesia and surgery are based on usual clinical practice. Adverse events will be recorded and followed if they are found to be serious or/and related to the vein puncture during blood collection. The occurrence of this kind of adverse events will be monitored. The rest of the adverse events will be treated as they are during the normal clinical practice.

### Patient and Public Involvement

Our interest in this area arose from the need for a new method to enhance risk stratification before surgery and guide intraoperative and early postoperative management of elderly patients. Parents will not play a role in recruitment. Participants will be sent a lay summary of the results when they are published. As a prospective observational cohort study, there was no intervention in our study design and exerted no additional burden on patients. But participants were given an opportunity to comment on the study in the final questionnaire.

### Dissemination

Dissemination of the findings will apply to local, national and international levels. Main results of the trial concerning clinical effectiveness will be submitted to a leading medical journal. Additional results of exploratory statistical analyses and qualitative/mixed-methods analyses of process data will be published in specialized international journals. Beyond journal publications, we will disseminate results through presentations at national and international conferences.

## Discussion

POD is a common complication in older surgical population with significant sequelae and associated burden on healthcare. Management of it can be categorized into risk stratification, risk reduction, early diagnosis, and treatment. With appropriate risk stratification, POD could then be managed through risk reduction measures and prophylactic interventions; it would also be possible to monitor high risk patients more closely and implement treatments more promptly. However, the multifactorial manner of this syndrome has made it hard to predict it. Because of the shortage of current risk prediction models and blood markers, we are trying to find new ways based on mitochondrial function.

In fact, mitochondrial function has been implicated and studied in numerous complex age-related diseases, including AD, diabetes, and atherosclerosis. And people have tried to expand clinical application of mitochondrial theory based on these findings. For example, resveratrol, an activator of NAD (+)-dependent deacetylase enzymes, has been shown to have beneficial effects on glucose metabolism and vascular function in animal models and patients (44, 45). In the perioperative period, there is few studies about mitochondrial function. Although BHI of monocytes has been proved to be a sensitive measure of oxidative stress in human and significantly depressed in patients undergoing cardiac surgery (35.36), there is still no clinical study connect it with the postoperative complications. Considering the pathophysiology of POD in aging patients, inflammation and oxidative stress act as the common root cause. This is likely the first study to find whether perioperative mitochondrial function of monocytes isolated from an individual’s blood can predict POD. There are some limitations to this protocol. Firstly, in order to ensure patient safety, patients with hemodynamic instability will be withdrawn from the study, that might cause a potential selection bias. Then, rarely, monocytes isolated from blood samples may not be sufficient for bioenergetic assessment. These patients will be excluded from analysis and the missing data will cause an exclusive bias. Thirdly, this is a single center study and the sample size is relatively small.

In conclusion, our study will analysis correlations between HBI of monocytes isolated from individual’s peripheral blood and delirium after major abdominal surgery in elderly patients during hospitalization. By exploring new and more effective methods to predict POD, we hope to identify high risk patients before surgery and develop perioperative strategies for them. Also, this project will advance the use of clinical study mitochondrial function during surgery.

## Data Availability Statement

The original contributions presented in the study are included in the article/supplementary material, further inquiries can be directed to the corresponding author/s.

## Ethics Statement

The studies involving human participants were reviewed and approved by the Ethics Committee on Biomedical Research, West China Hospital of Sichuan University, Sichuan, China (Chairperson Prof Shaolin Deng) on 11 June 2021 and registered at China clinical trial registry (ChiCTR2100047554). The patients/participants provided their written informed consent to participate in this study.

## Author Contributions

YZ, JL, and XH involved in design of the study and were active steering committee members for the study. MO contributed to the design and development of the protocol and was the statistical lead. All authors critically revised the manuscript, gave final approval of the manuscript and are accountable for the accuracy and integrity of the manuscript.

## Conflict of Interest

The authors declare that the research was conducted in the absence of any commercial or financial relationships that could be construed as a potential conflict of interest.

## Publisher’s Note

All claims expressed in this article are solely those of the authors and do not necessarily represent those of their affiliated organizations, or those of the publisher, the editors and the reviewers. Any product that may be evaluated in this article, or claim that may be made by its manufacturer, is not guaranteed or endorsed by the publisher.
